# Chronic Active Parietal Osteomyelitis Due to Salmonella typhi in a Patient with Sickle Cell Anemia

**DOI:** 10.4274/tjh.2017.0094

**Published:** 2017-12-01

**Authors:** Ahmad Antar, George Karam, Maurice Kfoury, Nadim El-Majzoub

**Affiliations:** 1 Almoosa Specialist Hospital, Department of Internal Medicine, Division of Hematology-Oncology, Al-Ahsa, Saudi Arabia; 2 Almoosa Specialist Hospital, Department of Neurosurgery, Al-Ahsa, Saudi Arabia; 3 Almoosa Specialist Hospital, Department of Diagnostic Radiology, Al-Ahsa, Saudi Arabia; 4 American University of Beirut Medical Center, Department of Pathology and Laboratory Medicine, Beirut, Lebanon

**Keywords:** Sickle cell anemia, Osteomyelitis, Salmonella typhi

## To The Editor,

Sickle cell disease (SCD) is a genetic disorder characterized by marked heterogeneity in clinical and hematologic severity, with musculoskeletal system manifestations being a major cause of morbidity and disability [1]. The increased susceptibility of SCD patients to infections, including osteomyelitis, has long been recognized with several mechanisms postulated including impaired splenic function, defects in complement activation, genetic factors, deficiencies in micronutrients, and the presence of infarcted or necrotic bone [2]. Salmonella is the most common cause of osteomyelitis in SCD, followed by Staphylococcus aureus and gram-negative enteric bacilli; this prevalence could be related to the fact that intravascular sickling of the bowel leads to patchy ischemic infarction [3,4]. The most common sites of osteomyelitis are the femur, tibia, or humerus. Patients usually present with acute onset of pain, swelling, and tenderness over the affected area in association with fever and elevated inflammatory markers. However, in some cases, osteomyelitis has atypical presentations with a more indolent course and often with abscess formation [5]. Here we present a 50-year-old female patient with sickle cell anemia (SCA) who developed parietal osteomyelitis with abscess formation and involvement of the dura due to Salmonella typhi, who was treated successfully by surgery followed by antibiotics.

**Figure 1:**
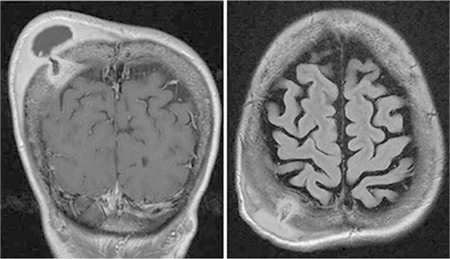
Magnetic resonance imaging of the brain demonstrating peripheral enhancement of the scalp abscess alongside noticeable enhancement of the adjacent soft tissues, pathological bone, and underlying pachymeningeal layer enhanced on a coronal T1W slice (left) with a clear intra-osseous edematous edema in keeping with osteomyelitic changes isolating a central bone sequestrum, communicating with a subperiosteal/subgaleal fluid collection on a fluid attenuated inversion recovery weighted slice (right).

A 50-year-old Saudi female patient living in the Eastern Province of Saudi Arabia, diagnosed with SCA (HgS: 78%) with occasional vaso-occlusive crisis and no sickle cell-related complications, presented to us with a 1-month history of a painless right parietal subgaleal collection increasing in size over time with no history of trauma and no fever or neurological manifestations. Laboratory testing revealed an elevated white blood cell count and a high estimated sedimentation rate level (125 mm/h). Magnetic resonance imaging of the brain revealed an osteolytic defect centered on the right parietal bone and sizable subgaleal complex collection (Figure 1). The patient underwent right parietal craniectomy with cranioplasty (removal of the right parietal subgaleal collection and the corresponding bone in addition to the invaded dura). Pathology of the specimen revealed a right parietal subgaleal abscess and right parietal bone chronic active osteomyelitis. Culture of the specimen grew Salmonella typhi.

The morbidity of chronic osteomyelitis combined with other complications of SCD decreases patients’ quality of life. Patients with SCD are more prone to osteomyelitis. The most common causative organism is Salmonella. The usual manifestations of osteomyelitis are pain, swelling, tenderness, and fever. However, like in our case, sometimes osteomyelitis presents late, as a more indolent process often with abscess formation and in unusual and more critical sites. Our case highlights the atypical presentation of osteomyelitis in a patient with SCD, which could cause devastating complications if not treated properly, early, and by a multidisciplinary team approach.
